# Structural Characterization of Maize SIRK1 Kinase Domain Reveals an Unusual Architecture of the Activation Segment

**DOI:** 10.3389/fpls.2017.00852

**Published:** 2017-05-26

**Authors:** Bruno Aquino, Rafael M. Couñago, Natalia Verza, Lucas M. Ferreira, Katlin B. Massirer, Opher Gileadi, Paulo Arruda

**Affiliations:** ^1^Structural Genomics Consortium, Universidade Estadual de CampinasCampinas, Brazil; ^2^Centro de Biologia Molecular e Engenharia Genética, Universidade Estadual de CampinasCampinas, Brazil; ^3^Structural Genomics Consortium, Nuffield Department of Clinical Medicine, University of OxfordOxford, United Kingdom; ^4^Departamento de Genética e Evolução, Instituto de Biologia, Universidade Estadual de CampinasCampinas, Brazil

**Keywords:** maize, receptor-like kinase, SIRK1, structure, ligand

## Abstract

Kinases are primary regulators of plant metabolism and excellent targets for plant breeding. However, most kinases, including the abundant receptor-like kinases (RLK), have no assigned role. SIRK1 is a leucine-rich repeat receptor-like kinase (LRR-RLK), the largest family of RLK. In *Arabidopsis thaliana*, SIRK1 (*At*SIRK1) is phosphorylated after sucrose is resupplied to sucrose-starved seedlings and it modulates the sugar response by phosphorylating several substrates. In maize, the *Zm*SIRK1 expression is altered in response to drought stress. In neither Arabidopsis nor in maize has the function of SIRK1 been completely elucidated. As a first step toward the biochemical characterization of *Zm*SIRK1, we obtained its recombinant kinase domain, demonstrated that it binds AMP-PNP, a non-hydrolysable ATP-analog, and solved the structure of *Zm*SIRK1- AMP-PNP co-crystal. The *Zm*SIRK1 crystal structure revealed a unique conformation for the activation segment. In an attempt to find inhibitors for *Zm*SIRK1, we screened a focused small molecule library and identified six compounds that stabilized *Zm*SIRK1 against thermal melt. ITC analysis confirmed that three of these compounds bound to *Zm*SIRK1 with low micromolar affinity. Solving the 3D structure of *Zm*SIRK1-AMP-PNP co-crystal provided information on the molecular mechanism of *Zm*SIRK1 activity. Furthermore, the identification of small molecules that bind this kinase can serve as initial backbone for development of new potent and selective *Zm*SIRK1 antagonists.

## Introduction

Kinases are thought to be key regulators of the mechanism by which plants respond to water restriction. Among kinases implicated in this process are some of the receptor-like kinases (RLK), which, at 610 members, comprise the largest kinase family in plants (Shiu and Bleecker, 2001). RLKs have also been implicated in innate immunity (Albert et al., 2010), pathogen response (Dodds and Rathjen, 2010), abiotic stress (Yang et al., 2010) and development and metabolism (De Smet et al., 2009). The largest sub-group of RLK possesses an extracellular domain containing leucine-rich repeats (LRR) (Shiu and Bleecker, 2001). These LRR-RLK are typically composed of a signal peptide, an extracellular domain with 1 to 30 LRR, a transmembrane domain and a cytoplasmic kinase domain. Crystal structures of LRR-RLK kinase domains revealed a canonical protein kinase fold and helped elucidate the cellular function of some LRR-RLK family members. For example, in the BIR2 structure access to the protein ATP-binding pocket was blocked by the β1 strand. Furthermore, changes in the amino acid sequence of BIR2 P-loop, a conserved region in active kinases, prevented this protein from coordinating ATP properly. Together, these observations provided structural reasons for the inability of BIR2 to bind ATP and to function as a transferase (Blaum et al., 2014). The crystal structure of another LRR-RLK, BRI1, helped rationalize the observation that phosphorylation of amino acids in the protein activation segment could regulate BRI1 activity and increased its substrate specificity (Bojar et al., 2014).

Sucrose-induced receptor kinase 1 (SIRK1) is a member of the LRR-RLK family (Niittyla et al., 2007). In Arabidopsis, SIRK1 (*At*SIRK1) is phosphorylated at residue S744*^At^*^SIRK1^ after sucrose resupply to sucrose-starved plants and seems to be part of a signaling cascade regulating protoplast swelling and water uptake (Wu et al., 2013). *At*SIRK1 is catalytically active and phosphorylates several substrates despite having amino acid substitutions at conserved positions important for kinase activity, including at the protein P-loop (GxGxxG; G to S), activation segment (HRDxKxxN; D to N) and DFG (DYC) motifs (Wu et al., 2013).

Much less is known about the *At*SIRK1 ortholog in maize, *Zm*SIRK1. Expression profiling data reveals that the gene coding for *Zm*SIRK1 is down-regulated under drought stress and up-regulated after re-watering (Zheng et al., 2010); and is mainly expressed in the base of developing leaves (Sekhon et al., 2011; Stelpflug et al., 2016).

We have a long-term aim of elucidating the function of *Zm*SIRK1 in drought stress by developing a chemical inhibitor that specifically targets its kinase domain. As a step toward this goal we cloned, expressed and purified the *Zm*SIRK1 kinase domain, identified several kinase inhibitors that bind to the enzyme and determined the first crystal structure of its kinase domain. We also identified small molecule kinase inhibitors that can bind to *Zm*SIRK1 and that might serve as starting points for the structure-guided development of more potent and selective inhibitors.

## Materials and Methods

### RNA Extraction and cDNA Synthesis

Total RNA was extracted from leaves of 15-day-old maize B73 plants using PureLink RNA mini kit (Life Technologies, Carlsbad). Briefly, leaves were collected, immediately frozen in liquid nitrogen, and then powdered using a mortar. Two hundred mg of frozen powder was added to 1 mL of TRIzol (Invitrogen, Carlsbad). After 10 min incubation at room temperature with gentle shaking, 200 μL of chloroform was added and the mixture was homogenized under vigorous agitation using a vortex. After centrifugation for 15 min at 12,000 ×*g* at 4°C, the upper aqueous phase was collected and applied to a PureLink RNA (Life Technologies, Carlsbad) column. The column was washed with Wash buffer I followed by Wash buffer II and eluted in RNAse-free water as per the manufacturer’s instructions. cDNA was synthesized using SuperScript III reverse transcriptase (Invitrogen, Carlsbad). Two microgram of total RNA were incubated with oligo dT20 and dNTP for 5 min at 65°C and then cooled on ice. Buffer, DTT and SuperScript enzyme were added and incubated for 60 min at 50°C. The enzyme was inactivated for 15 min at 70°C and the cDNA stored at -20°C until use.

### ZmSIRK1 cDNA Cloning and Protein Expression in *Escherichia coli*

Four different N-terminal truncations of the *Zm*SIRK1 kinase domain (residues His623-Ser1045; Ser737-Ser1045; Ser751-Ser1045; and Ser760-Ser1045) were cloned into pNIC28a-Bsa4 (Savitsky et al., 2010). Forward primers used were: sirk1-H623-F (TACTTCCAATCCATGCATTGGAAGATCAGTAGCTGGAAA AG); sirk1-S737-F (TACTTCCAATCCATGTCAGTTGTGTTCA CGGCTGAAG); sirk1-S751-F (TACTTCCAATCCATGTCT CCTGATAAACTGGTTGGGG); sirk1-S760-F (TACTTCCA ATCCATGTCTTGTGCCCCTGCTGAG) and reverse primer used was sirk1-S1045-R (TATCCACCTTTACTGTCACGAC GATAAGGACAAGAGATC). The cDNA synthesized from total RNA of maize B73 leaves was used as a template for PCR. After PCR amplifications, amplicons were treated with T4-DNA polymerase for ligase-independent cloning (LIC) (Savitsky et al., 2010). Positive clones for the four constructs were transformed into BL21(DE3)-R3-pRARE2 and grown in 500 μL in 96 × 1 mL well-block. After overnight growth, individual cultures were diluted 1:100 in LB medium with kanamycin (50 μg/mL) and incubated at 37°C with shaking. When the OD_600_ reached 2, the temperature was lowered to 18°C, 0.1 mM of IPTG was added and the culture incubated overnight with shaking. Cultures were then centrifuged at 3,500 ×*g* for 20 min at 4°C and lysed using 200 μL of Lysis buffer (50 mM HEPES pH 7.5; 500 mM NaCl; 10% glycerol; 10 mM imidazole; 500 μM TCEP; 0.1% dodecyl maltoside; 1 mM MgCl_2_; 1:200 protease inhibitor; 0.5 mg/mL lysozyme; 50 units/mL benzonase). After lysis, cultures were centrifuged at 3,500 ×*g* for 10 min at 4°C and the supernatant was incubated for 1 h at 18°C with 50 μL of Ni^2+^-sepharose beads (GE Healthcare, Uppsala). After washing with wash buffer (50 mM HEPES pH 7.5; 500 mM NaCl; 10% glycerol; 30 mM imidazole; 500 μM TCEP), purified proteins were eluted with 50 μL of 50 mM HEPES pH 7.5; 500 mM NaCl; 10% glycerol; 300 mM imidazole; 500 μM TCEP. Expression and solubility were verified in 12.5% SDS-PAGE (Laemmli, 1970).

### Large-Scale Protein Production and Purification

Vector pNIC28a-Bsa4 harboring construct *Zm*SIRK1^737-1045^ was transformed into competent BL21(DE3)-R3-pRARE2 *Escherichia coli* cells. Pre-culture was grown in 20 ml of LB media grown overnight and then inoculated into 1.5 L of Terrific Broth at 37°C until OD_600_ of 1.5. The culture was cooled down to 18°C, 0.2 mM of IPTG was added to the medium and growth resumed overnight. Cells were collected by centrifugation (15 min at 7,500 ×*g* at room temperature). Cell pellet was suspended in 2× binding buffer (1× binding buffer is 500 mM HEPES; 500 mM NaCl; 5% glycerol; 10 mM imidazole; 1 mM TCEP) with protease inhibitors (1:200) and frozen at -80°C until use. Suspended cell pellets were thawed and sonicated for 9 min at 4°C (5 s ON; 10 s OFF; Amp 30%). One ml of 5% polyethyleneimine (pH 7.5) was added per 30 ml of lysate and the sample was centrifuged at 53,000 ×*g* for 45 min at 4°C. The supernatant was loaded onto an IMAC column (5 ml HisTrap FF Crude) and washed in binding buffer with 30 mM imidazole. Recombinant protein was eluted with elution buffer (binding buffer with 300 mM imidazole). To remove the 6xHis-tag, eluted protein was incubated with TEV protease and the tag removed using nickel beads. The protein solution was loaded onto a size exclusion HiLoad 16/60 Superdex 200pg (GE) column equilibrated in a gel filtration buffer (binding buffer without imidazole). Fractions of 1.8 mL were collected and verified for protein purity in a 12.5% SDS-PAGE gel. Purified fractions were pooled together and stored at -80°C.

### Crystallization, Data Collection, Structure Determination and Refinement

A mixture containing equimolar quantities of adenylyl-imidodiphosphate (AMP-PNP) and MgCl_2_ was added to purified *Zm*SIRK1^737-1045^ (850 μM) at threefold molar excess. The solution was incubated on ice for approximately 30 min. The mixture was centrifuged at 20,000 ×*g* for 10 min at 4°C prior to setting up 150 nl volume sitting drops at three ratios of the protein-inhibitor complex to reservoir solution (2:1, 1:1, or 1:2). Crystallization experiments were performed at 20°C. The best-diffracting crystals grew under the conditions described in **Table 1**, first identified from the Morpheus Crystallization screen (Gorrec, 2015). Crystals were cryoprotected in reservoir solution supplemented with 20–25% glycerol before flash-freezing in liquid nitrogen for data collection. Diffraction data were collected at the Advanced Photon Source (APS), integrated using XDS (Kabsch, 2010) and scaled using AIMLESS from the CCP4 software suite (Winn et al., 2011). Molecular replacement (MR) was performed with Phaser (McCoy et al., 2007) using the kinase domain of BAK1 interacting RLK 2 (BIR2) as the search model (PDB ID 4L68) (Blaum et al., 2014). Automated model building was performed with Buccaneer (Cowtan, 2006) following density modification with Parrot (Zhang et al., 1997). Automated refinement was performed in Buster (Global Phasing Ltd.). Coot (Emsley et al., 2010) was used for manual model building and refinement. Structure validation was performed using MolProbity (Chen et al., 2010). Structure factors and coordinates (**Table 1**) have been deposited in the PDB.

**Table 1:** Crystallographic data.

Data collection	
X-ray source	APS 24-ID-C
Wavelength (Å)	0.9790
Space group	P2_1_2_1_2_1_
Cell dimensions (Å)	
*a, b, c* (Å)	53.4 74.5 79.7
Resolution (Å)^∗^	39.9–2.3
No. of unique reflections^∗^	57,012 (1,084)
*R*_merge_ (%)^∗^	4.9 (55.1)
Mean I/σI^∗^	14.8 (2.6)
Completeness (%)^∗^	97.0 (98.4)
Redundancy^∗^	4.0 (4.1)
CC_1/2_**^‡^**	0.99 (0.91)
**Refinement**	
Resolution range (Å)	39.8–2.3
*R*/*R*_free_ (%)	18.2/21.1
Mean B-factor (Å)	66.9
r.m.s.d. bond lengths (Å)^§^	0.010
r.m.s.d. bong angles (degrees)^§^	1.06
**Ramachandran plot statistics (%)**	
Preferred regions	98.2
Outlier	0.0
**PDB ID**	5UV4
**Crystallization conditions**	12,5% PEG1000; 12,5% PEG3350; 12,5% MPD; 0.02M of each D-glucose, D-mannose, D-galactose, L-fucose, D-xylose and *N-*acetyl-D-glucosamine; 0.1 M MOPS/HEPES-Na pH7.5

*^∗^The highest resolution shell is shown in parenthesis. ^‡^CC_1/2_ indicates the percentage of correlation between intensities of random half data sets (Karplus and Diederichs, 2012). ^§^r.m.s.d., root mean squared deviation from ideal geometry.*

### Differential Scanning Fluorimetry (DSF)

Thermal stabilization (DSF) assays were performed essentially as described (Niesen et al., 2007), with the following modifications. Purified *Zm*SIRK1^737-1045^ was screened against a library of 378 structurally diverse and cell-permeable ATP-competitive kinase inhibitors library from Selleckchem (Houston, TX, United States; catalog No. L1200). DSF experiments were performed in a 384-well plate format. Each well contained 25 μL of 1 μM kinase in DSF buffer (100 mM K-phosphate, 150 mM NaCl, 10% glycerol, pH 7.5) and the Protein Thermal Shift dye at the recommended concentration of 1:1000 (Applied Biosystems; the composition of the buffer and the dye solutions are not disclosed). Compounds (10 mM) in DMSO were added to 10 μM final concentration (0.1% final DMSO). Plates were sealed using optically clear films and transferred to a QuantStudio 6 qPCR instrument (Applied Biosystems, Singapore). Fluorescence intensity data were acquired in a temperature gradient from 25 to 95°C at a constant rate of 0.05°C/s and protein melting temperatures were calculated based on a Boltzmann function fitting to experimental data, as implemented in the Protein Thermal Shift Software (Applied Biosystems, Singapore). Protein in 0.1% DMSO was used as a reference. Compounds displaying a positive temperature shift (ΔTm) of 2°C or higher compared to the control were considered positive.

### Isothermal Calorimetry (ITC)

Solutions containing 50 μM purified *Zm*SIRK1^737-1045^ protein (cell) and 1 mM AMP-PNP (injectant) were prepared in ITC buffer (50 mM K-phosphate; 500 mM NaCl, 5% glycerol; 1 mM TCEP; 50 mM MgCl_2_). For ITC measurements, 50 μM of each compound (cell) and 500 μM purified *Zm*SIRK1^737-1045^ (injectant) were prepared in ITC buffer without Mg^2+^. ITC experiments were performed in MicroCal AutiITC200 (GE, Northampton) and titrations were carried out at 25°C with a stirring speed of 750 rpm and 300 s between each 2 μL injection. Controls with buffer and ligand alone were performed to verify the dilution heat and were subtracted from protein-ligand data. Data were analyzed in Origin 7 software package.

### Phylogenetic Analyses

The kinase domain of *Zm*SIRK1 was used to search related proteins within UniProt Knowledge database (*e*-value of 10^-4^; limit: 1,000 sequences). To verify the prevalence of putative phosphorylation sites within the activation segment of *Zm*SIRK1 homologs we developed a script in java. The script performed a search within the identified proteins and reported on the presence/absence of the conserved DYS motif. If the DYS motif was present, then the script reported on the presence or absence of possible phosphorylation sites (Tyr, Ser, or Thr residues) within SIRK1 activation segment.

## Results

### Heterologous Expression and Purification of *Zm*SIRK1 Kinase Domain

**FIGURE 1:**
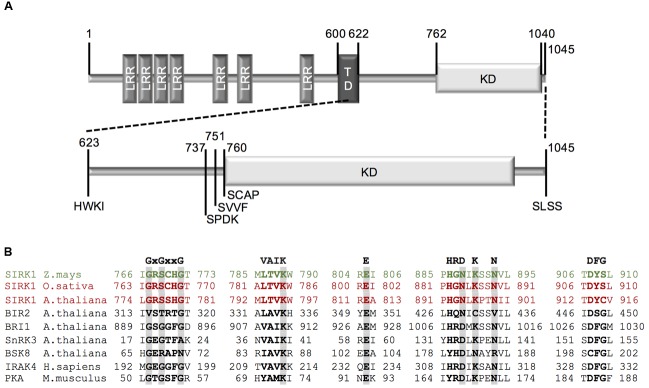
**Cloning strategy for *Zm*SIRK1. (A)** Schematic representation of full-length *Zm*SIRK1. The extracellular domain (aa 1–600) contains seven leucine-rich repeats (LRR) and is separated from the intracellular domain by a transmembrane domain (TD) (aa 601–622). The intracellular domain (aa 623–1045) contains a kinase domain (KD) (aa 762–1040). Numbers represents the amino acid position and letters the amino acid sequence from the beginning of each construct used for recombinant protein expression. **(B)** Amino acid sequence alignment of conserved motifs within *Zm*SIRK1 kinase domains with other protein kinases. GxGxxG: glycine-rich loop; VAIK: motif harboring the catalytic lysine; E: conserved glutamate in helix αC; HRDxKxxN: catalytic loop containing the catalytic aspartate; DFG: DFG motif.

Constructs harboring the kinase domain of *Zm*SIRK1 were tested for recombinant expression in *E. coli* to obtain high yields of soluble protein. Four N-terminal truncations of the *Zm*SIRK1 cytoplasmic domain were designed and cloned into pNIC28a-Bsa4 (**Figure 1A** and Supplementary Figures S1A,B). Large-scale protein expression using construct *Zm*SIRK1^737-1045^ yielded ∼110 mg of ≥98% pure recombinant protein using 1.5 L of culture (Supplementary Figures S1C–E). The identity of recombinant *Zm*SIRK1 kinase domain was verified by mass spectrometry (data not shown).

### Crystallization and Overall Structure of *Zm*SIRK1 Kinase Domain

**FIGURE 2:**
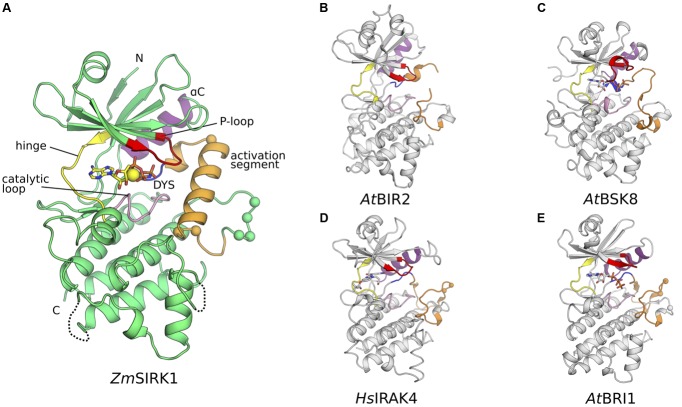
**Co-crystal structure of *Zm*SIRK1 bound to AMP-PNP. (A)** Cartoon representation of the *Zm*SIRK1-AMP-PNP structure. Protein regions were colored as follows: glycine-rich (P-)loop – red; hinge region – yellow; catalytic loop -pink; DYS (more commonly DFG) motif – blue; activation segment – orange; αC – purple. Other protein regions in green. AMP-PNP is shown in stick and Mg^2+^ ion as a yellow sphere. The Cαs from putative phosphorylation sites within and immediately C-terminal of *Zm*SIRK1 activation segment are shown as spheres. **(B–E)** Cartoon representation of *Zm*SIRK1 closest structural neighbors. **(B)** Crystal structure of *At*BIR2 (PDB ID 4L68). **(C)** Crystal structure of *At*BSK8 (PDB ID 4I92). **(D)** Crystal structure of *Hs*IRAK4 (PDB ID 2NRY). **(E)** Crystal structure of *At*BRI1 (PDB ID 5LPB). Protein regions colored as in **(A)**. If present, ligands are represented as sticks and the Cαs from known phosphorylation sites as spheres.

Crystallization conditions for purified *Zm*SIRK1 kinase domain were identified using commercially available sparse matrix crystallization screens, resulting in large *Zm*SIRK1-AMP-PNP co-crystals (**Table 1** and Supplementary Figure S1F). The crystal structure of *Zm*SIRK1 kinase domain bound to the non-hydrolysable ATP analog, AMP-PNP, was solved by molecular replacement using the kinase domain of *At*BIR2, an RLK from Arabidopsis, as the search model (PDB ID 4L68; 35% sequence identity) (Blaum et al., 2014) (**Table 1**). Overall, the *Zm*SIRK1 kinase domain has the canonical kinase topology, with an N-terminal lobe composed mostly of β-sheets connected by a flexible linker (hinge region) to a C-terminal lobe predominantly composed of α-helices (**Figure 2A**). In the crystal structure, AMP-PNP is bound to the ATP-binding pocket of the *Zm*SIRK1 kinase domain, located in a cleft between N- and C-terminal lobes. Electron density maps allowed placement of most residues in *Zm*SIRK1. The final model consisted of residues 744–972, 977–1000, and 1009–1045. Omitted regions of the polypeptide chain in the final model located to the protein N-termini or to loop regions in the C-terminal lobe, which are likely to be disordered due to flexibility.

### The Activation Segment of *Zm*SIRK1 Adopts an Unusual Conformation

We compared the structure of *Zm*SIRK1 against those in the PDB using the DALI server (Holm and Rosenstrom, 2010). This search identified the kinase domains of *At*BIR2 (BAK1-interacting RLK 2) and *At*BSK8 (brassinosteroid signaling kinase 8) as the closest structural neighbors to *Zm*SIRK1. Both *At*BIR2 and *At*BSK8 are known to be pseudo-kinases and to lack catalytic activity (Grutter et al., 2013; Blaum et al., 2014). The next closest structural neighbors to *Zm*SIRK1 were the human protein kinase IRAK4 (interleukin-1 receptor-associated kinase 4) (Wang et al., 2006) and the kinase domain of the brassinosteroid insensitive membrane receptor from Arabidopsis (*At*BRI1) (Bojar et al., 2014) (**Figures 2A–E**).

**FIGURE 3:**
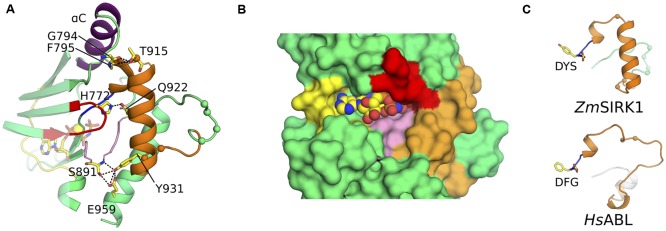
***Zm*SIRK1 activation segment is highly structured. (A)** Cartoon representation of *Zm*SIRK1 around the activation segment. Residues taking part in the stabilization of this segment are shown in stick. Possible hydrogen bonds are represented as dashed lines. **(B)** Surface representation of *Zm*SIRK1 nucleotide-binding site showing that the protein activation segment (orange) occludes the region normally available to interact with the target peptide. AMP-PNP is shown as spheres. **(C)** The 3_10_ helix immediately C-terminal of *Zm*SIRK DYS motif (top) is also seen in inactive state mammalian kinase domains (bottom – human ABL; PDB ID 2G1T). The Cαs from putative phosphorylation sites in *Zm*SIRK1 and from known phosphorylation sites in *Hs*ABL are shown as spheres.

Compared to its closest structural neighbors, the most notable feature of *Zm*SIRK1 kinase domain is the unusual architecture of its activation segment (highlighted in orange in **Figure 2**). The activation segment of kinases extends from the DFG (^907^DYS^909^ in *Zm*SIRK1) to the APE (^933^PPE^935^ in *Zm*SIRK1) motifs. In most protein kinases, this region is composed of a flexible loop. The position of this loop controls the activations state of the enzyme. Kinases in a fully active state have activation segments in an extended or open conformation. By contrast, in the inactive kinase conformation, the position of the activation segment occludes the peptide-binding site. In *Zm*SIRK1, the activation segment is highly structured. The region immediately C-terminal to the DYS motif forms a single turn 3_10_ helix that packs against αC. This helical turn is followed by a short loop region and an α-helix (P916*^Zm^*^SIRK1^-L929*^Zm^*^SIRK1^). The position and organization of the *Zm*SIRK1 activation segment are stabilized by hydrogen bonds to residues from both lobes of the protein (**Figure 3A**). Residue Gln922 within the α-helix hydrogen bonds to residues in both the glycine-rich (H772*^Zm^*^SIRK1^) and catalytic loops (G887*^Zm^*^SIRK1^). Moreover, Y931*^Zm^*^SIRK1^, located at the C-terminal end of the activation loop, hydrogen bonds to catalytic loop residue S891*^Zm^*^SIRK1^ and to E959*^Zm^*^SIRK1^ from α-helix F (subdomain IX in PKA). These structures partly occlude the kinase ATP-binding site and peptide substrate-binding pocket, hallmarks of the inactive state (**Figure 3B**). An equivalent 3_10_ helix has been observed in the inactive state of mammalian kinases, such as ABL, SRC, and CDK2 (De Bondt et al., 1993; Schindler et al., 1999; Xu et al., 1999; Levinson et al., 2006). However, in these proteins, the 3_10_ helix is followed by a mostly disordered region, whereas in *Zm*SIRK1 we observe a short loop followed by an α-helix (**Figure 3C**).

### Conservation of Putative Phosphorylation Sites within *Zm*SIRK1 Activation Domain

**FIGURE 4:**
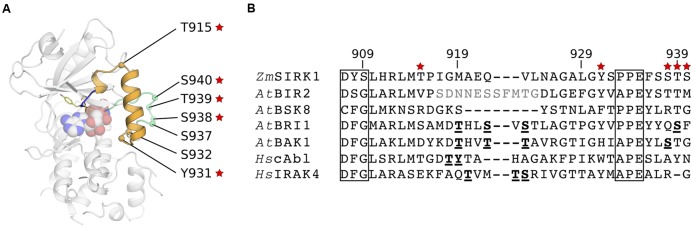
**Conservation of possible phosphorylation sites within *Zm*SIRK1 activation segment. (A)** Cartoon representation of *Zm*SIRK1 highlighting the position of possible phosphorylation sites within (orange) and C-terminal (green) of the protein activation segment. Red stars indicate sites with high (>80%) conservation within SIRK1 homologs. **(B)** Structure-based sequence alignment of the activation segment in *Zm*SIRK and its closest structural neighbors. Conserved kinase domain motifs (commonly DFG and APE) are boxed. Residues in bold and underlined are known phosphorylation sites. Residues in gray were not visible in the structure.

In most kinases, phosphorylation of regulatory Ser/Thr residues within the activation segment stabilizes the active state conformation of the kinase by counteracting an Arg residue within the HRD motif. Within the *Zm*SIRK1 activation segment, there are three putative phosphorylation sites (**Figure 4A**). The first, T915*^Zm^*^SIRK1^, is located in the loop between the helical elements within the activation segment. The next two possible phosphorylation sites, Y931*^Zm^*^SIRK1^ and S932*^Zm^*^SIRK1^, are found immediately after the α-helix. There are also three other possible phosphorylation sites immediately C-terminal to the activation segment (residues 937–940). Inspection of electron density maps did not suggest these residues are phosphorylated in the *Zm*SIRK1-AMP-PNP crystal structure. Moreover, the HRD motif in *Zm*SIRK1 is degraded and the positively charged Arg is replaced with a Gly. In some of *Zm*SIRK1 closest structural neighbors, both from plant and mammals, phosphorylation of Ser/Thr residues within the activation domain is required for activity (Hantschel and Superti-Furga, 2004; Wang et al., 2005; Cheng et al., 2007; Kuglstatter et al., 2007; Yan et al., 2012) (**Figure 4B**).

We reasoned that if these putative phosphorylation sites are involved in protein activation, they should be conserved amongst SIRK1 homologs. A search within UniProt using the kinase domain of *Zm*SIRK1 revealed 124 proteins, exclusively from plants, bearing the unusual DYS motif present in maize SIRK1 (sequence identities between 29 and 98%). We then interrogated these sequences for the presence of putative phosphorylation sites (Thr/Tyr/Ser residues) at equivalent positions within and immediately C-terminal of *Zm*SIRK1 activation segment – +6, +22, +23, and +29–31 positions C-terminal from the DYS motif. We found that 119 out of 124 proteins with a DYS motif in Uniprot displayed a Thr residue at position +6, which locates to the spacer loop between the 3_10_ and α helices within *Zm*SIRK1 activation segment. We also found that all 124 proteins displayed a Tyr residue at positions +22 – located immediately C-terminal to the α-helical segment in *Zm*SIRK1; in addition to having a Thr residue at position +6. Conservation at positions +29 (Ser), +30 (Ser/Thr), and +31 (Ser/Thr) was also high (ranging from 80 to 90%), whereas sites at +23 and +28 were markedly less conserved (<21%). Overall, co-conservation of the most prevalent residues occurred in 79 out of the 124 identified proteins (∼64%). These analyses also identified 209 plant SIRK1 homologs with the DYC motif seen in *At*SIRK1. Conservation of possible phosphorylation sites within these proteins is similar, but not identical to those identified above for *Zm*SIRK1 (Supplementary Table S1).

### *Zm*SIRK1 Adopts an Inactive, DFG-in/αC-Out Conformation

In many kinases, the positions of αC and of the DFG motif in the active and inactive states are also different. In the active conformation, αC swings inward toward the ATP-binding pocket (αC-in conformation) and facilitates the formation of a salt bridge between a conserved glutamic residue within αC (E805*^Zm^*^SIRK1^) and the catalytic lysine (K789*^Zm^*^SIRK1^). This interaction is key for enzyme activity. By contrast in the inactive αC-out conformation observed for a few mammalian proteins, this conserved glutamic residue is solvent-exposed and makes a salt link to an Arg residue from the kinase activation segment (Levinson et al., 2006). The interaction of the conserved aspartic residue within the DFG motif to the Mg^2+^ ion is also critical for transphosphorylase activity. This interaction is only possible in the so-called DFG-in conformation. In this configuration, the hydrophobic residue within the DFG motif takes part in a series of hydrophobic contacts that stabilizes the active state conformation of the enzyme (R-spine) (Kornev et al., 2006). In an active kinase, both DFG-in and αC-in configurations are usually present.

**FIGURE 5:**
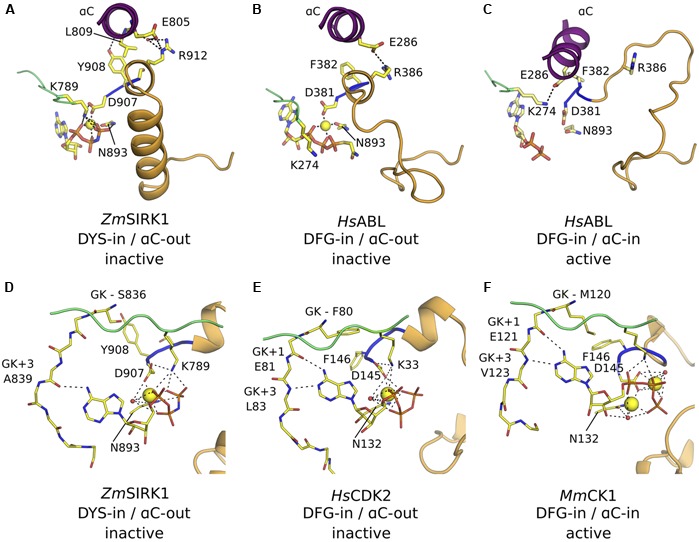
***Zm*SIRK1 adopts an inactive DFG-in/αC-out conformation. (A)** Cartoon representation of *Zm*SIRK1 around αC, DYS motif and activation segment showing residues (stick) and interactions (dashed lines) that stabilize the inactive conformation in this protein. **(B)** Human proteins, such as *Hs*ABL (PDB ID 2G1T), can also adopt the DFG-in/αC-out conformation. **(C)** Phosphorylation of regulatory sites (not shown) within *Hs*ABL stabilizes the active (DFG-in/αC-in) conformation. **(D)** Details of *Zm*SIRK1 interaction with Mg^2+^ ion and AMP-PNP showing the unusual Mg^2+^ ion-coordination, ribose puckering and adenine ring interactions in *Zm*SIRK1-AMP-PNP co-structure. Possible hydrogen bonds are shown as dashes. **(E)** Inactive state human proteins, such as CDK2 (PDB ID 1HCK) can also display unusual Mg^2+^ ion coordination and sugar puckering. **(F)** Mg^2+^ ion coordination and nucleotide interaction for the prototypical active state kinase domain of *Mus musculus* (*Ms*)CK1 (PDB ID 1ATP). Residues in the hinge region are annotated relative to the gatekeeper residue (GK, GK+1, GK+3).

The kinase domain in the *Zm*SIRK1-AMP-PNP co-structure displays a DYS-in conformation and the side chain atoms from D907*^Zm^*^SIRK1^ within this motif participates in the octahedral coordination of the Mg^2+^ ion bound to AMP-PNP. However, coordination of the divalent ion is unusual (see below) and the position of Y908*^Zm^*^SIRK1^ in the *Zm*SIRK1-AMP-PNP co-structure prevents assembly of the hydrophobic residues in the kinase domain core characteristic of active state proteins. The αC in *Zm*SIRK1 is away from the ATP-binding site (αC-out) and the side chain of E805*^Zm^*^SIRK1^ is solvent exposed and makes a hydrogen bond to R912*^Zm^*^SIRK1^ in the activation segment. Moreover, the hydroxyl group from Y908*^Zm^*^SIRK1^ hydrogen bonds to the main chain carbonyl group from L809*^Zm^*^SIRK1^ in αC, further stabilizing the observed “twisted-out” configuration of this secondary structure (**Figure 5A**). The DFG-in/αC-out out configuration has been observed for a few mammalian kinases, such as ABL, SRC, and CDK2 (**Figure 5B**), but thus far not in any plant kinase domains. For the mammalian kinases, phosphorylation of activations segment Ser/Thr residues can stabilize the active, DFG-in/αC-in conformation (**Figure 5C**).

### Interaction of *Zm*SIRK1 with AMP-PNP

In the co-crystal structure, the ATP analog adopts an unusual conformation. The ribose is found in a C2^′^-endo conformation, compared to the more commonly observed 3^′^-endo puckering. The position of the β-phosphate is also quite distinct and, consequently, so are the position and coordination of the Mg^2+^ ion (**Figure 5D**). The conformation observed for AMP-PNP here is similar to that in the inactive state structure of the mammalian protein CDK2 (Schulze-Gahmen et al., 1996) (**Figure 5E**) and contrasts with that observed for kinases in their active state, especially in what concerns the position of the nucleotide phosphate β (**Figure 5F**).

The position of the ATP-analog within the *Zm*SIRK1 binding pocket is also unusual. The two lobes of a kinase domain are connected by a flexible “hinge” region. In *Zm*SIRK1, the hinge region spans residues S836-L844*^Zm^*^SIRK1^. In general, ATP and its analogs are anchored to the kinase domain via two hydrogen bonds. The adenosine N6 amine and N1αα imine interact with the backbone of residues located at +1 and +3 positions relative to the so-called gatekeeper residue (GK, S836*^Zm^*^SIRK1^), respectively. These interactions ensure the nucleotide sits deep within the enzyme binding pocket. However, in *Zm*SIRK1-AMP-PNP co-crystals, the N6 amine from the ligand interacts with the more solvent-exposed residue at GK+3 (A839*^Zm^*^SIRK1^). This is the only hydrogen bond between ligand and hinge residues. As a result, AMP-PNP sits closer to the solvent than it is normally observed for other kinases.

In most kinases, proper orientation of ATP phosphate groups within the kinase nucleotide-binding pocket relies on the close contacts afforded by glycine residues within the protein P-loop and is crucial for catalysis. As for other plant RLKs, the P-loop of *Zm*SIRK1 does not display the canonical consensus sequence (GxGxxG). In *Zm*SIRK1, the second Gly within this motif is replaced with S770*^Zm^*^SIRK1^. In the *Zm*SIRK1-AMP-PNP co-structure, the side chain from this serine residue hydrogen bonds to the γ-phosphate in AMP-PNP. The bulkier side chain of S770*^Zm^*^SIRK1^ also prevents the nucleotide β-phosphate to interact closely with main chain groups from the P-loop. Instead, the phosphate groups from AMP-PNP interact mostly with protein residues in the protein C-terminal domain, opposite to the N-terminal P-loop.

### Ligand Interaction

**FIGURE 6:**
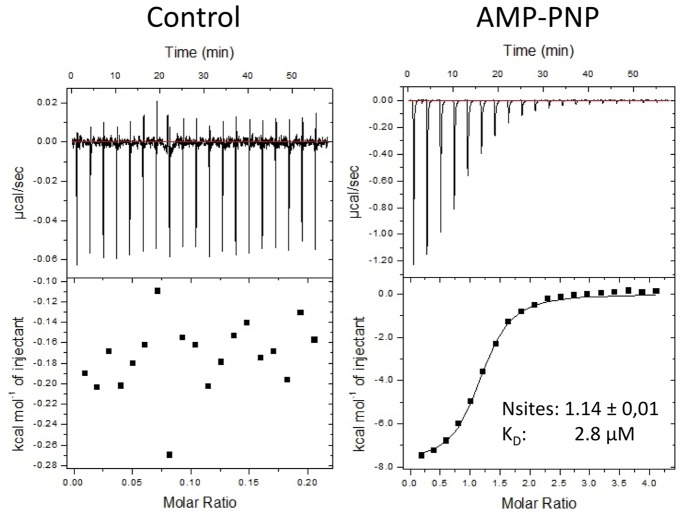
***Zm*SIRK1 binds to AMP-PNP in solution.** Isothermal titration calorimetry (ITC) measurements for the interaction between AMP-PNP and *Zm*SIRK1. Protein (50 μM of *Zm*SIRK1^737-1045^) was titrated to AMP-PNP (1 mM) in ITC buffer (50 mM K-phosphate; 500 mM NaCl, 5% glycerol; 1 mM TCEP; 50 mM MgCl_2_). Y-axis in left (control) and right (AMP-PNP) panels are not in the same scale.

In Arabidopsis, the expression of *At*SIRK1 is induced by sucrose and the enzyme phosphorylates an aquaporin regulating water content inside cells. Identification of potent antagonists of *Zm*SIRK1 may help elucidate the role of this kinase during drought stress. Some of the closest structural homologs of *Zm*SIRK1 are pseudo-kinases, including BIR2 which does not bind ATP (Blaum et al., 2014). We thus determined if purified recombinant *Zm*SIRK1 could bind the non-hydrolysable ATP analog AMP-PNP using isothermal titration calorimetry (ITC). The ITC results indicated that the protein can bind the AMP-PNP in a magnesium-dependent manner with a *K*_D_ of 2.8 μM (**Figure 6**).

We next used thermal denaturation assays (DSF) to identify small molecules within a 378-compound collection of chemically diverse human kinase inhibitors that could interact with *Zm*SIRK1. We identified three compounds with temperature shifts, ΔTms, over 2.0°C, an arbitrary cut-off for a positive hit in this experiment (**Table 2**). DSF results for these compounds were further confirmed using ITC. The compounds PP242, INK 128 and PP121 bound *Zm*SIRK1 with *K*_D_s of 0.46, 1.76, and 1.99 μM, respectively (**Table 2** and Supplementary Figure S2).

**Table 2:** Thermal shift (DSF) and ITC data for selected *Zm*SIRK1^737-1045^ ligands.

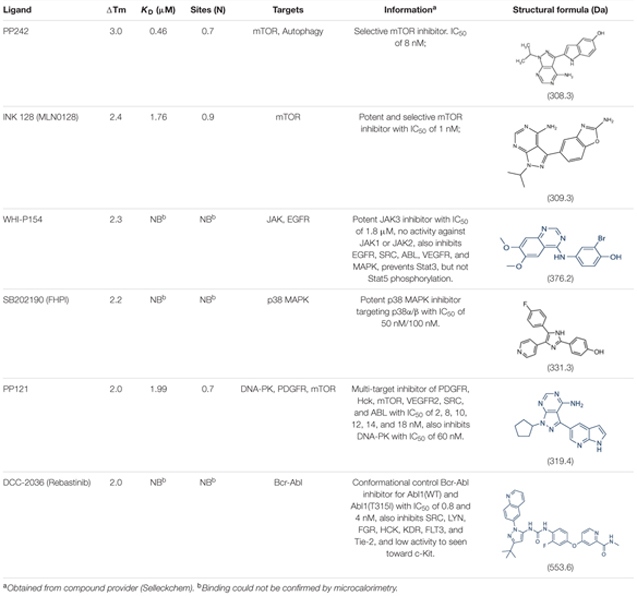

The *Zm*SIRK1-ATP-PNP co-structure showed a partly closed ATP-binding site. To assess if compounds identified by DSF and verified by ITC would be able to fit into the ATP-binding pocket of *Zm*SIRK1, we performed a simple rigid-body docking of PP121 to the *Zm*SIRK1-AMP-PNP co-structure. This exercise used the available co-structure of PP121 bound to the human kinase domain SRC (PDB ID 3EN4) (Apsel et al., 2008) and suggested PP121 can fit in the ATP-binding pocket of *Zm*SIRK1 and occupies the bottom of this cavity with few steric clashes to protein residues (Supplementary Figure S3). PP242 and INK 128 are closely related to PP121 (**Table 2**) and we expect they could also fit without major clashes in the nucleotide-binding pocket of *Zm*SIRK1. We have not yet been able to obtain co-crystal structures with these synthetic ligands.

## Discussion

Extensive structural characterization of mammalian kinase domains has captured a number of proteins in both active and inactive states. Phosphorylation of regulatory sites within the kinase activation segment stabilizes the closed, active state of the protein – a conserved activating mechanism observed in most protein kinases. Activation of plant protein kinases also requires phosphorylation of regulatory sites within the activation segment. For example, *At*BRI1 activation segment must be phosphorylated for the kinase to be active (Wang et al., 2005). However, the structural details of how plant kinases switch from inactive to active states have not yet been completely characterized. The structure of the *Zm*SIRK1 kinase domain obtained here suggests a possible activation mechanism for this and related plant kinases.

The activation segment of *Zm*SIRK1 adopts an unusual conformation – a single-turn 3_10_ helix connected to an α-helix by a short loop. The α-helix within the activation segment occupies the putative peptide substrate-binding pocket. The protein is in a DYS-in/αC-out inactive conformation, reminiscent of that observed for the inactive state of mammalian proteins, such as SRC, ABL, and CDK2 (De Bondt et al., 1993; Schindler et al., 1999; Xu et al., 1999; Levinson et al., 2006). Activation of these kinases requires phosphorylation of regulatory sites, mostly located at the proteins activation segment. *Zm*SIRK1 has several conserved, putative phosphorylation sites within and immediately C-terminal to its activation segment. Some of these putative phosphorylation sites in SIRK1 are structurally equivalent to known phosphorylation sites in *At*BRI1 that stabilize the activation segment of this protein in an extended, active conformation (Tang et al., 2008; Bojar et al., 2014). It is thus possible that phosphorylation of regulatory sites within *Zm*SIRK1 activation segment elicits the large conformational changes required for this region of the protein to adopt an extended conformation. These structural movements would re-position the short helical turn within the activation segment and allow αC to swing inwards toward the ATP-binding site. The *Zm*SIRK1-AMP-PNP co-crystal suggests that ligand binding alone is not sufficient to elicit these conformational changes.

The use of small molecules to modulate protein function has been extremely successful in assigning gene function in animal cells (Arrowsmith et al., 2015). For human proteins, such as bromodomains and protein kinases, several potent and selective chemical probes are available (Bain et al., 2007; Shortt et al., 2017). A similar approach has only started to be applied to plants. For example, glycogen synthase kinase 3 (GSK3) activity is required for brassinosteroid signaling. A loss-of-function mutation in the gene encoding GSK3 BIN2, a known negative regulator of the brassinosteroid signaling pathway, is not sufficient to activate this pathway. Polyploidy and genome duplication is a common phenomenon in plants and gene redundancy can overcome the deleterious effects of having a non-functional copy of a particular gene (Comai, 2005). However, the small molecule bikinin, an inhibitor of BIN2 and other GSK3, can activate the brassinosteroid signaling pathway in a hormone-independent manner (Vert and Chory, 2006; De Rybel et al., 2009). Thus, small molecule inhibitors targeting closely related members of the same kinase family may be used to overcome the inherent genetic redundancy of plants.

The evolutionary distances and lifestyle differences between plants and animals are likely to be reflected in both sequence and structure of plant kinases. The primary sequence of SIRK1 differs from that of mammalian kinases in key regions of the protein, such as the activation segment, DFG/APE motifs and the P-loop. In many plant kinases, degradation of these conserved motifs has led to an inability to bind nucleotides (Blaum et al., 2014). However, we show here that *Zm*SIRK1 can bind to nucleotides and identified related synthetic compounds with similar chemotypes that can bind to *Zm*SRIK1 with low micromolar affinity, an indication that these molecules interact with a specific site within the protein, and that chemical libraries designed for human kinases will generate hits for some members of the plant kinome.

## Conclusion

Here we report the first crystal structure of a SIRK1 kinase domain. Our results further suggest activation mechanism are conserved in both plant and mammalian protein kinases and that the plant enzymes can interact with compounds designed for their mammalian counterparts. Further work is required to elucidate the impact of phosphorylation on the structure and transphosphorylase activity of *Zm*SIRK1.

## Accession

Structure factors and coordinates were deposited in the PDB (PDB ID 5UV4).

## Author Contributions

BA and PA conceived the project; BA executed all the experiments; NV, helped identify the drought stress response of *Zm*SIRK1. RC helped prepare the protein crystal, collected the X-ray diffraction data and solved the protein structure; RC and BA analyzed the structural data; BA, RC, and PA wrote the paper. KM, OG, and PA helped coordinate the project. LF performed the phylogenetic analyses. All authors reviewed the manuscript.

## Conflict of Interest Statement

The authors declare that the research was conducted in the absence of any commercial or financial relationships that could be construed as a potential conflict of interest.
