# Supplementary material for: Structural Characterization of Maize SIRK1 Kinase Domain Reveals an Unusual Architecture of the Activation Segment
Source: Front Plant Sci. 2017 May 26;8:852. doi: 10.3389/fpls.2017.00852 (PMC5445127; doi:10.3389/fpls.2017.00852)
Supplement: Supplementary file 1 [file Data_Sheet_1.DOCX]

**Structural characterization of maize SIRK1 kinase domain reveals an unusual architecture of the activation segment**

Bruno Aquino^1^, Rafael M. Couñago^1,2^, Natalia Verza^1,2^, Lucas M. Ferreira^1^, Katlin B. Massirer^1,2^, Opher Gileadi^1,3^ and Paulo Arruda^1,2,4^

^1^Structural Genomics Consortium, Universidade Estadual de Campinas - UNICAMP, 13083-886, Campinas, SP, Brazil.

^2^Centro de Biologia Molecular e Engenharia Genética, Universidade Estadual de Campinas (UNICAMP), 13083-875, Campinas, SP, Brazil.

^3^Structural Genomics Consortium, Nuffield Department of Clinical Medicine, University of Oxford, OX3 7DQ, UK.

^4^Departamento de Genética e Evolução, Instituto de Biologia, Universidade Estadual de Campinas (UNICAMP), 13083-970, Campinas, SP, Brazil.

Bruno Aquino - brunoaquino_14@hotmail.com

Rafael Couñago - rafaelcounago@gmail.com

Lucas Ferreira - lucas@lmferreira.com

Katlin Massirer - katlinbm@gmail.com

Opher Gileadi - opher.gileadi@sgc.ox.ac.uk

Running title: Crystallographic structure of maize SIRK1 receptor kinase

Corresponding author:

Paulo Arruda

Tel: +55 - 19 - 35211137

Fax: +55 - 19 - 35211089

E-mail: [parruda@unicamp.br](mailto:parruda@unicamp.br)

**SUPPLEMENTARY MATERIAL**

**SUPPLEMENTARY FIGURE LEGENDS**

**Supplementary Figure S1.** Cloning, expression and purification of *Zm*SIRK1 kinase domain. **(A)** Agarose gels showing SIRK1 amplicons using cDNA prepared from total RNA of young leaves from maize B73. Amplicons were named using the first and last amino acid in the corresponding recombinant protein. **(B)** Small-scale expression test of *Zm*SIRK1 kinase domain constructs resolved in 12,5% SDS-PAGE. TL – total lysate from BL21(DE3)-R3-pRARE2 cells overexpressing the correspond construct; S - soluble fraction after cell lysate clarification by centrifugation; yellow arrow indicates the overexpressed band in size expected for each construct. **(C)** SDS-PAGE (12.5%) showing fractions from a typical IMAC purification of recombinant *Zm*SIRK1^737-1045^. TL - total lysate from BL21(DE3)-R3-pRARE2 cells overexpressing construct *Zm*SIRK1^737-1045^; S - soluble fraction after cell lysate clarification by centrifugation; FT - flow-through fraction from IMAC column; W - wash fraction from IMAC column (30 mM imidazole); E - eluted fraction from IMAC column (300 mM imidazole). Following IMAC, the eluted fraction was treated with TEV protease to remove the N-terminal His-tag introduced as part of the cloning strategy (lane TEV+). **(D)** TEV protease-treated *Zm*SIRK1^737-1045^ was injected onto a gel filtration column and fractions were analyzed in 12.5% SDS-PAGE. **(E)** Purified *Zm*SIRK1^737-1045^. After gel filtration, samples were polled together and stored at -80°C. Expected molecular weight for His-tag free *Zm*SIRK1^737-1045^ is 34.2 kDa. (**F**) Typical co-crystals of *Zm*SIRK1^737-1045^ - AMP-PNP growing in a sitting (150 nl) drop set up. The largest rod crystal at the center is ~0.5 mm in length.

**Supplementary Figure S2. Interaction of *Zm*SIRK1 to small molecule ligands.** (**A-F**) Thermal denaturation **(**DSF) (left panels) and microcalorimetry (ITC) results (right panels). For DSF experiments (left panels), *Zm*SIRK1 was incubated with 10-fold molar excess of the indicated compounds (blue traces) and subjected to a thermal denaturation gradient in the presence of an environment-dependent fluorescent dye. Protein containing DMSO only (red traces) was used as a control to calculate differences (ΔTm) in temperature stabilization. For the indicated compounds, upper panels show fluorescence melt curves and lower panels show derivative melt curves. For the microcalorimetry experiments 50 µM of compound was titrated with 500 µM of *Zm*SIRK1^737-1045^ protein in ITC buffer (50 mM K-phosphate; 500 mM NaCl, 5% glycerol; 1 mM TCEP). Upper panels show raw data and lower panels show integrated heat data.

**Supplementary Figure S3.** ***Zm*SIRK1 ATP-biding site can accommodate identified small molecules.** Compound PP121 (in light green sticks) was docked to *Zm*SIRK1-AMP-PNP co-structure following superposition of human SRC-PP121 (PDB ID 3EN4) to *Zm*SIRK1-AMP-PNP. AMP-PNP is shown as lines. The gatekeeper (S836), the catalytic lysine (K789), N893 and those residues in the hinge region are shown as lines. The protein activation segment is shown as cartoon (orange).

**SUPPLEMENTARY TABLES**

| **SUPPLEMENTARY TABLE S1. Conservation of putative phosphorylation sites within the activation segment of SIRK1 proteins** | | | | | | | | | | | | | | | | | | | | | |
| --- | --- | --- | --- | --- | --- | --- | --- | --- | --- | --- | --- | --- | --- | --- | --- | --- | --- | --- | --- | --- | --- |
| Motif  (n) | Position C-terminal to DYS / DYC motif | | | | | | | | | | | | | | | | | | | | |
|  | +6 | | | +10 | | | +22 | | | +23 | | | +29 | | | +30 | | | +31 | | |
|  | S | T | Y | S | T | Y | S | T | Y | S | T | Y | S | T | Y | S | T | Y | S | T | Y |
| DYS (124) | 1 | 118 | 0 | 0 | 89 | 0 | 0 | 0 | 124 | 26 | 0 | 0 | 97 | 2 | 0 | 65 | 43 | 0 | 106 | 5 | 0 |
| DYC (209) | 16 | 182 | 0 | 0 | 129 | 0 | 0 | 0 | 207 | 25 | 0 | 0 | 43 | 4 | 0 | 137 | 0 | 18 | 40 | 1 | 0 |

**SUPPLEMENTARY FIGURES**

**Supplementary Figure S1**


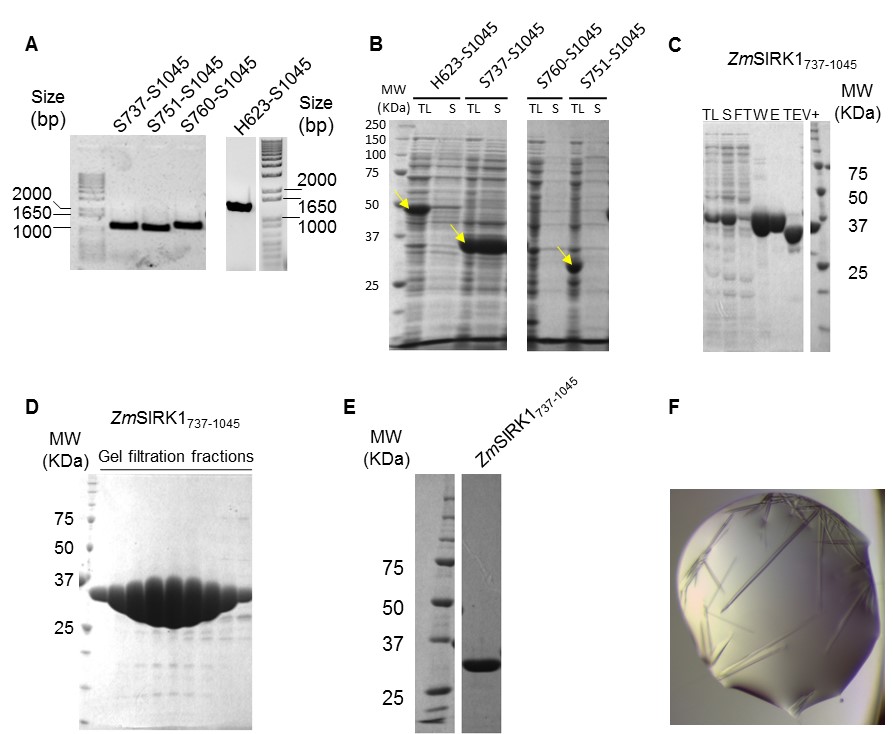


**Supplementary Figure S2**

**
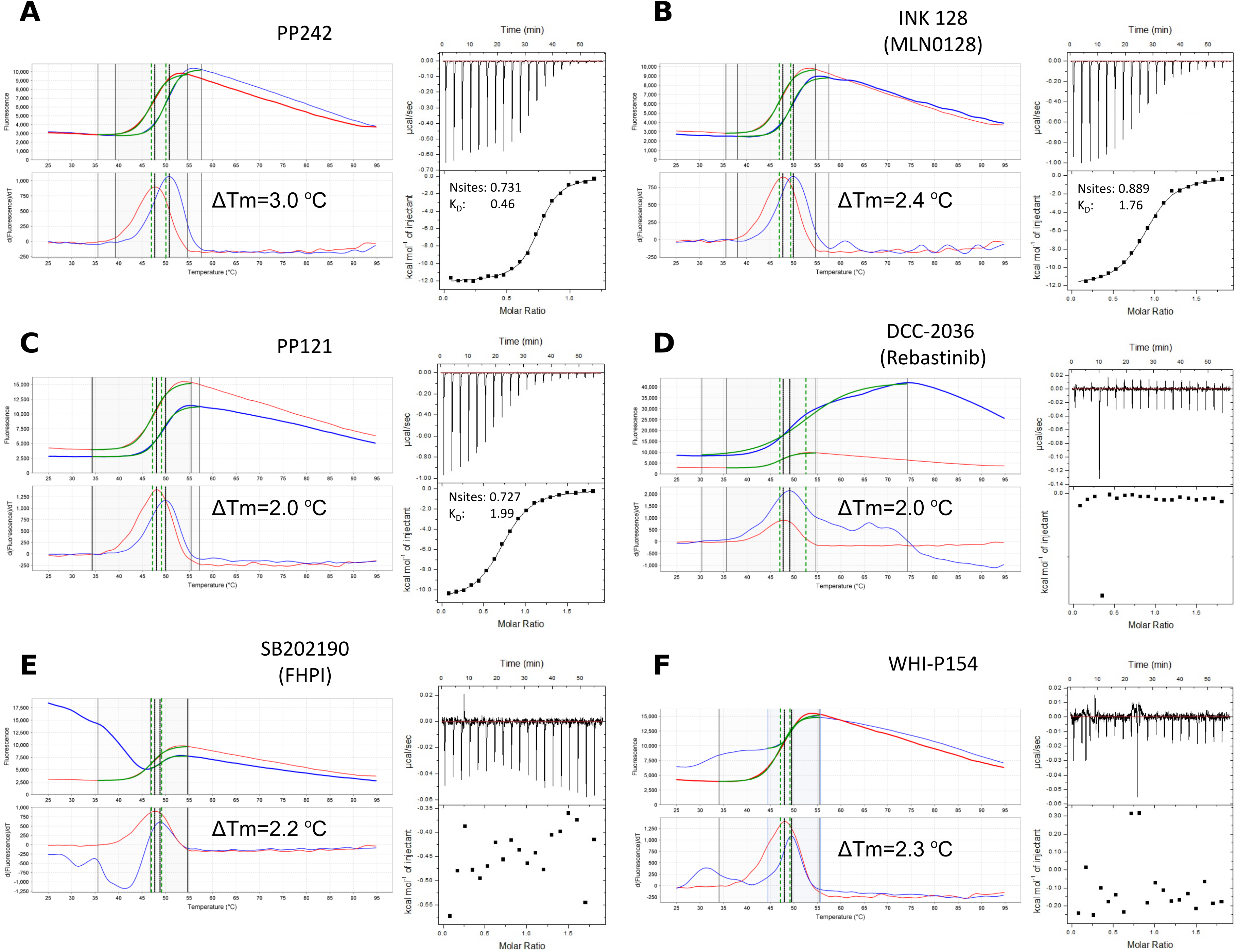
**

**Supplementary Figure S3**

**
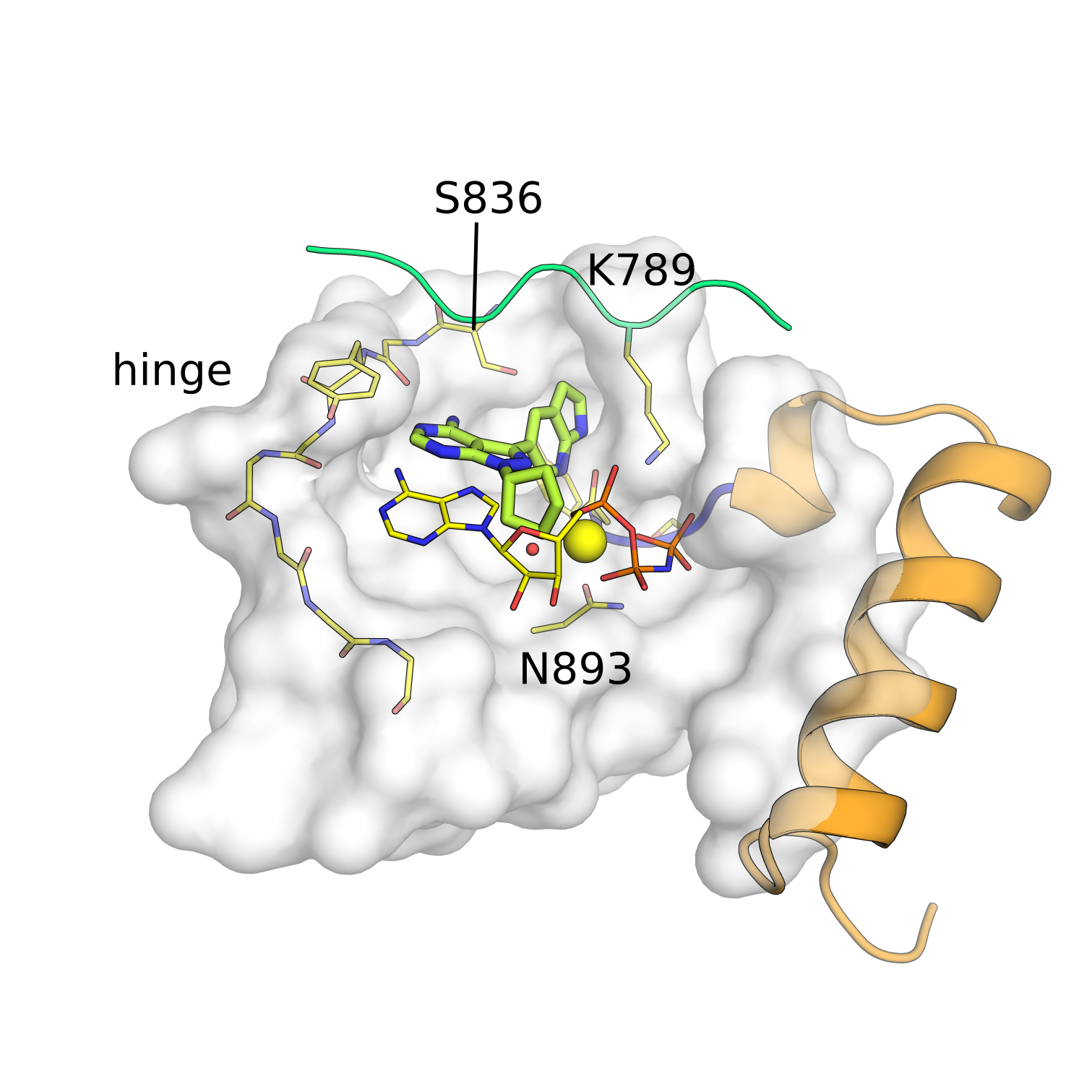
**
